# Differences in Gut Microbiome in Hospitalized Immunocompetent vs. Immunocompromised Children, Including Those With Sickle Cell Disease

**DOI:** 10.3389/fped.2020.583446

**Published:** 2020-11-12

**Authors:** Sindhu Mohandas, Vijaya L. Soma, Thi Dong Binh Tran, Erica Sodergren, Tresa Ambooken, David L. Goldman, George Weinstock, Betsy C. Herold

**Affiliations:** ^1^Division of Pediatric Infectious Diseases, The Children's Hospital at Montefiore, Bronx, NY, United States; ^2^Albert Einstein College of Medicine, Bronx, NY, United States; ^3^Division of Infectious Diseases, Children's Hospital Los Angeles, Keck School of Medicine, University of Southern California, Los Angeles, CA, United States; ^4^The Jackson Laboratory for Genomic Medicine, Farmington, CT, United States

**Keywords:** microbiome, clostridioides difficile, sickle cell disease, dysbiosis, immunocompromised and healthy subjects

## Abstract

**Background:** Gut microbial diversity and composition play important roles in health. This cross-sectional study was designed to test the hypothesis that hospitalized children who may be relatively immunocompromised (IC), defined as those with cancer, sickle cell disease (SCD), transplantation, or receiving immunosuppressive therapy) would have decreased microbial diversity, increased *Clostridioides difficile* colonization and different species composition compared to non-immunocompromised (Non-IC) children admitted to the same pediatric unit.

**Methods:** A stool sample was obtained within 72 h of admission to a single unit at The Children's Hospital at Montefiore, Bronx, NY from March 2016 to February 2017 and the microbiome assessed by 16S rRNA sequencing. *C. difficile* colonization was assessed by glutamate dehydrogenase antigen and toxin polymerase chain reaction assays.

**Results:** Stool samples were obtained from 69 IC (32 SCD, 19 cancer, 9 transplantation and 9 other) and 37 Non-IC patients. There were no significant differences in microbial alpha diversity and *C. difficile* colonization comparing IC vs. non-IC patients. Lower alpha diversity, however, was independently associated with the use of proton pump inhibitors or antibiotics, including prophylactic penicillin in patients with SCD. Differences in specific species abundances were observed when comparing IC vs. non-IC patients, particularly children with SCD. Non-IC patients had increased abundance of commensals associated with health including *Alistipes putredinis, Alistipes ihumii, Roseburia inulinivorans, Roseburia intestinalis*, and *Ruminococcus albus* (*p* < 0.005).

**Conclusions:** Antibiotics and proton pump inhibitors, which were more commonly used in IC children, were identified as risk factors for lower microbial diversity. Non-IC patients had higher abundance of several bacterial species associated with health. Longitudinal studies are needed to determine the clinical significance of these differences in gut microbiome.

## Introduction

The gastrointestinal (GI) microbiome is comprised of a highly diverse community of bacteria, fungi, protozoa and viruses that play critical roles in physiology, metabolism, health, and disease. More than 10^14^ microorganisms may be found in the intestine and the composition and diversity are impacted by diet, exposure to antibiotics and other environmental factors. Six bacterial phyla (*Firmicutes, Proteobacteria, Bacteroidetes, Fusobacteria, Actinobacteria*, and *Verrucomicrobia*) dominate the gut of healthy adults (1). Changes in microbial composition are associated with immune dysregulation and have been linked to multiple diseases including inflammatory bowel disease (IBD), obesity, autoimmune diseases, cancer, and infections (2–5). A loss of diversity may promote growth of pathogenic bacteria including toxin-producing *Clostridioides (C.) difficile* (6).

Maintenance of gut microbial diversity may prevent colonization and/or overgrowth of pathogenic bacteria by several mechanisms. For example, commensal bacteria may promote epithelial cells to produce antimicrobial peptides, which can inhibit growth of pathogens and reduce the risk of bacterial translocation from the gut to the systemic circulation (7). An increase in Enterococci or Enterobacteriaceae and a concomitant decrease in commensal anaerobic microbiota in the stool are associated with a significantly increased risk of bloodstream infections with the same bacterial species in hematopoietic cellular transplant (HCT) and cancer patients (8). These findings have led to speculation that real-time monitoring of the gut microbiome in IC populations may be of value in identifying patients at increased risk for future bacteremia and selecting empiric therapy pending culture identification. The gut microbiome may also influence response to cancer immunotherapies, engraftment following transplantation, and risk of graft-vs.-host disease (9, 10).

There are fewer studies evaluating the microbiome in pediatric patients and, specifically, at the time of hospitalization, and no studies comparing immunocompromised (IC) and non- immunocompromised (non-IC) children. To address this knowledge gap, we conducted a cross-sectional study of the gut microbiome in children admitted to a single unit in a pediatric hospital over a 1 year period to test the hypothesis that “IC” children, defined as those with diseases associated with immune suppression including cancer, HCT, solid organ transplantation (SOT) and sickle cell disease (SCD), would have decreased microbial diversity as well as higher rates of asymptomatic *C. difficile* colonization, a biomarker of decreased diversity, compared to non-IC children hospitalized on the same unit. These differences could contribute to adverse outcomes.

## Methods

### Study Population

The study was conducted on a single inpatient unit at the Children's Hospital of Montefiore in Bronx, New York, from March 2016 to February 2017. The unit includes patients with cancer, SCD, and HCT or SOT recipients. Low infectious risk patients are also hospitalized on this unit. The Albert Einstein College of Medicine-Montefiore Institutional Review Board approved the study.

All patients < 22 years of age were invited to participate in the study. Patients were excluded if they had *C. difficile* infection currently or within the preceding 2 months or had been previously enrolled in the study on a prior admission. The electronic medical record was reviewed for demographic data, medical and surgical history, prior hospitalizations, history of diarrhea including *C. difficile* colitis, antibiotic exposures within the previous 3 months, and other medications. Participants were requested to provide a stool sample within 72 h of admission. Stool samples were transported to the laboratory, immediately refrigerated, and subsequently divided into aliquots within 48 h and stored at −70°C.

### Sequencing of Bacterial 16S rRNA Gene

Metagenomic DNA was extracted from stool samples using a PowerSoil® DNA Isolation Kit (11). The V1 through V3 hyper-variable regions (V1-V3) of 16S rRNA genes were amplified from the metagenomic DNA using primers 27F and 534R (27F: 5'- AGAGTTTGATCCTGGCTCAG-3′ and 534R: 5'-ATTACCGCGGCTGCTGG-3′). The oligonucleotides containing the 16S primer sequences also contained an adaptor sequence for Illumina MiSeq platform as well as one of 240 tag sequences unique to each sample. A barcode sequence unique to each sample is embedded within each of the forward and reverse oligonucleotides used to create the amplicons (dual tags). The uniquely barcoded amplicons from multiple samples were pooled and sequenced on the Illumina MiSeq sequencing platform using a V3 2 × 300 sequencing protocol. Illumina's software was used for initial processing of all the raw sequencing data. One mismatch in primer and zero mismatch in barcodes are applied to assign read pairs to the appropriate sample within a pool of samples. Barcode and primers were removed from the reads. Reads were further processed by removing primers using Trimmomatic v0.32 with the commands HEADCROP:20, TRAILING:10, MINLEN:100 (12) joining paired-end sequences using the FLASH algorithm (13) and removing chimeric sequences with UChime (14). The cleaned, assembled amplicons were the input data for analysis.

### *C. difficile* Testing

Samples were batched and analyzed for *C. difficile* glutamate dehydrogenase (GDH) and toxin A/B by enzyme immunoassay (EIA) (*C. diff quik chek complete*® Techlab) followed by toxin B PCR. (Xpert® *C. difficile/* Epi*)*. Colonization was defined as having a positive stool GDH assay; toxigenic colonization was defined as positive GDH plus a positive EIA or toxin B PCR assay in patients without diarrhea.

### Statistical Methodology

Metadata analyses were performed using Stata/SE 14.2 and Graphpad Prism (version 7). Chi-square test of association with the Pearson value was used to compare categorical variables between IC and non-IC patients and subpopulations. Logistic regression was used to calculate odds ratios. Multiple comparative analyses were performed using the Tukey's multiple comparisons test. All *P* values were 2-sided and used a 5% level of significance.

A sampling average depth of about 37,600 reads per sample of a total of 104 samples was used for microbiome downstream analysis using Phyloseq package (15). The Shannon Diversity Index was calculated as Σ piln(pi), where pi presents the proportional abundance of species. The non-parametric Wilcoxon and Kruskal-Wallis rank sum-tests were used for differential diversity or abundance between two or more groups, respectively, and corrected for multiple comparisons by Benjamini-Hochberg procedure. Beta diversity was analyzed using the Bray-Curtis distance for community abundance and Jaccard distance for community presence/absence. The among-group differences were determined using the permutational multivariate analysis of variance by the distance matrices (ADONIS) and the analysis of group similarities (ANOSIM). These tests compare the intragroup distances to the intergroup distances in a permutation scheme and then calculate a *p*-value. These functions are implemented in the Vegan package (16). For all permutation tests, we used 999 permutations by default setting in the Vegan package.

Random forest package was used to predict operational taxonomic units (OTUs) that differentiated groups. Based on the construction of multiple decision trees according to the bagging approach, each tree was built independently from a boostrap sampling. Using the mean decrease accuracy criterion, the classification was performed and identified the most reliable and relevant OTUs, this method ranks factors based on their ability to distinguish different groups by considering the complex interrelationship within a group. Principal coordinate analysis (PCoA) plots, boxplots and heatmaps were generated for graphical analysis by using Phyloseq, ggplot2 and ComplexHeatmap packages, respectively.

## Results

### Clinical and Demographic Features of Study Population

During the 12-month study period, 106 unique patients who met inclusion criteria provided a stool sample within 72 h of admission. Sixty-nine patients were classified as IC, including 19 with cancer, 32 with SCD, 3 HCT and 6 SOT recipients (4 liver and 2 renal), and 9 with other chronic illnesses associated with immune suppression. Thirty-seven patients were classified as non-IC (Supplementary Table 1). Clinical and demographic data are summarized in Table 1. Age of patients ranged from 0.01 to 20.84 years with a mean of 6.07 years. There was a trend for the IC cohort (mean age 5.71 years) to be older than the non-IC cohort (mean age 4.65 years) (*p* = 0.06). The IC patients were significantly more likely to be currently receiving antibiotics (*p* = 0.02), have received antibiotics within the last 3 months (*p* < 0.001) and to have had at least one prior hospitalization within the previous 3 months (*p* = 0.008). IC patients were also significantly more likely to be receiving a proton pump inhibitor (PPI) (*p* = 0.02) (Table 1). There were no differences in *C. difficile* colonization rates between the IC and non-IC cohorts. The majority of colonizing isolates were toxin B positive.

**Table 1:** Demographics and clinical characteristics of participants.

	**IC (*n* = 69)**	**Non IC (*n* = 37)**	***P*-value**
Age in years [mean (SD)]	8.3 (5.71)	4.65 (5.69)	0.06
Gender (Male: Female)	38:31	18:19	0.52
Race (Black: Hispanic: Other)	38:20:11	11:20:6	0.03
Antibiotics in last 3 months [n (%)]	43 (62%)	9 (24%)	< 0.001
Antibiotics during current admission [n (%)]	49 (71%)	18 (48%)	0.02
Hospitalization within last 3 months [n (%)]	33 (48%)	8 (22%)	0.008
Proton pump inhibitor use	15 (22%)	2 (5%)	0.02
*C. difficile* colonization (GDH) [n (%)]	16 (23%)	7 (19%)	0.61
Toxin B *+ C. difficile* by PCR [n (%)]	9/16 (56%)	5/7 (71%)	0.58

### Differences in Microbial Diversity and Predominant Species

**Figure 1:**
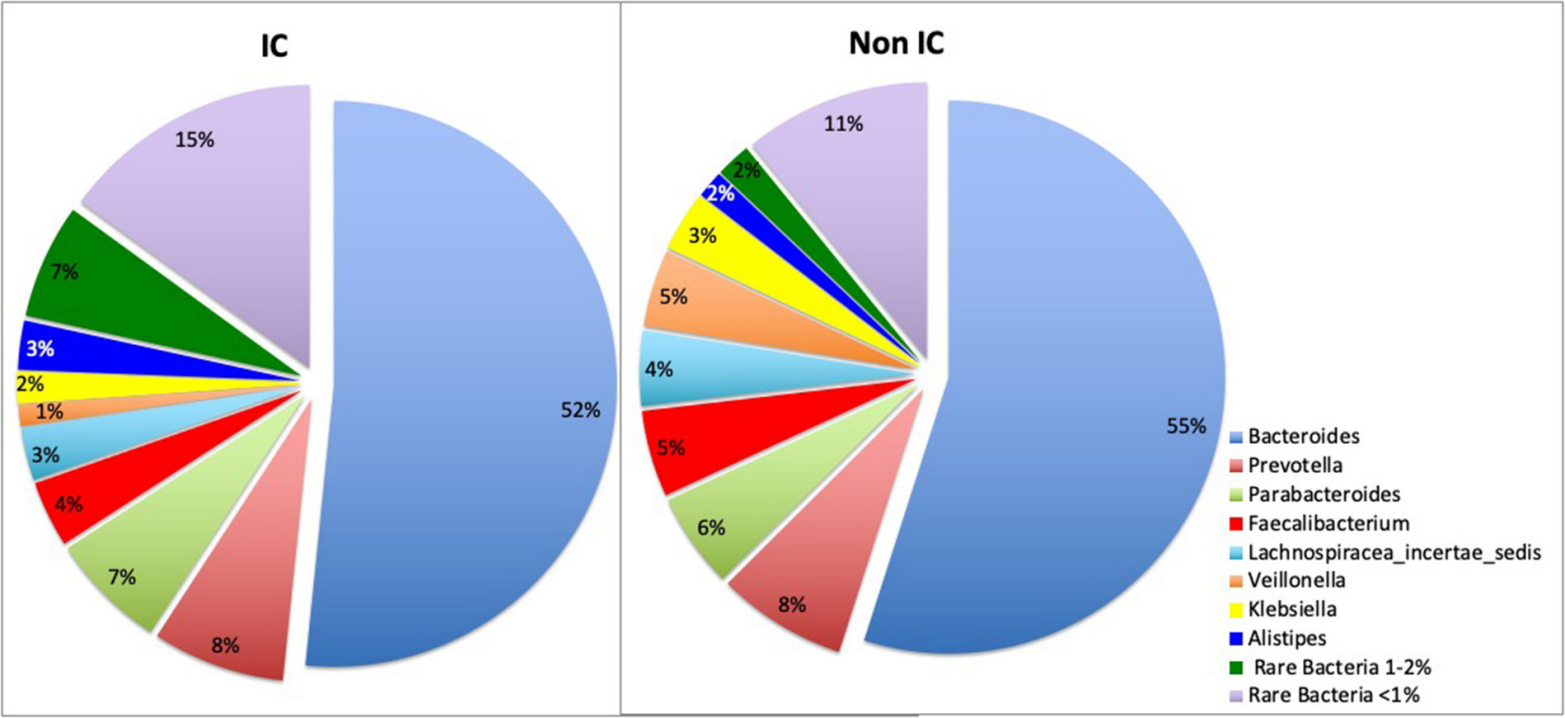
Pie charts represent total percentage read counts in the IC and non-IC groups for the corresponding color-coded genera with major genera including *Bacteroides, Prevotella, Parabacteroides, Faecalibacterium, Lachnospiracea_incertae_sedis, Veillonella, Klebsiella, Allistipes*. Other minor genera were regrouped as “rare bacteria comprising 1–2%” and “rare bacteria comprising < 1%” of total read counts.

**Figure 2:**
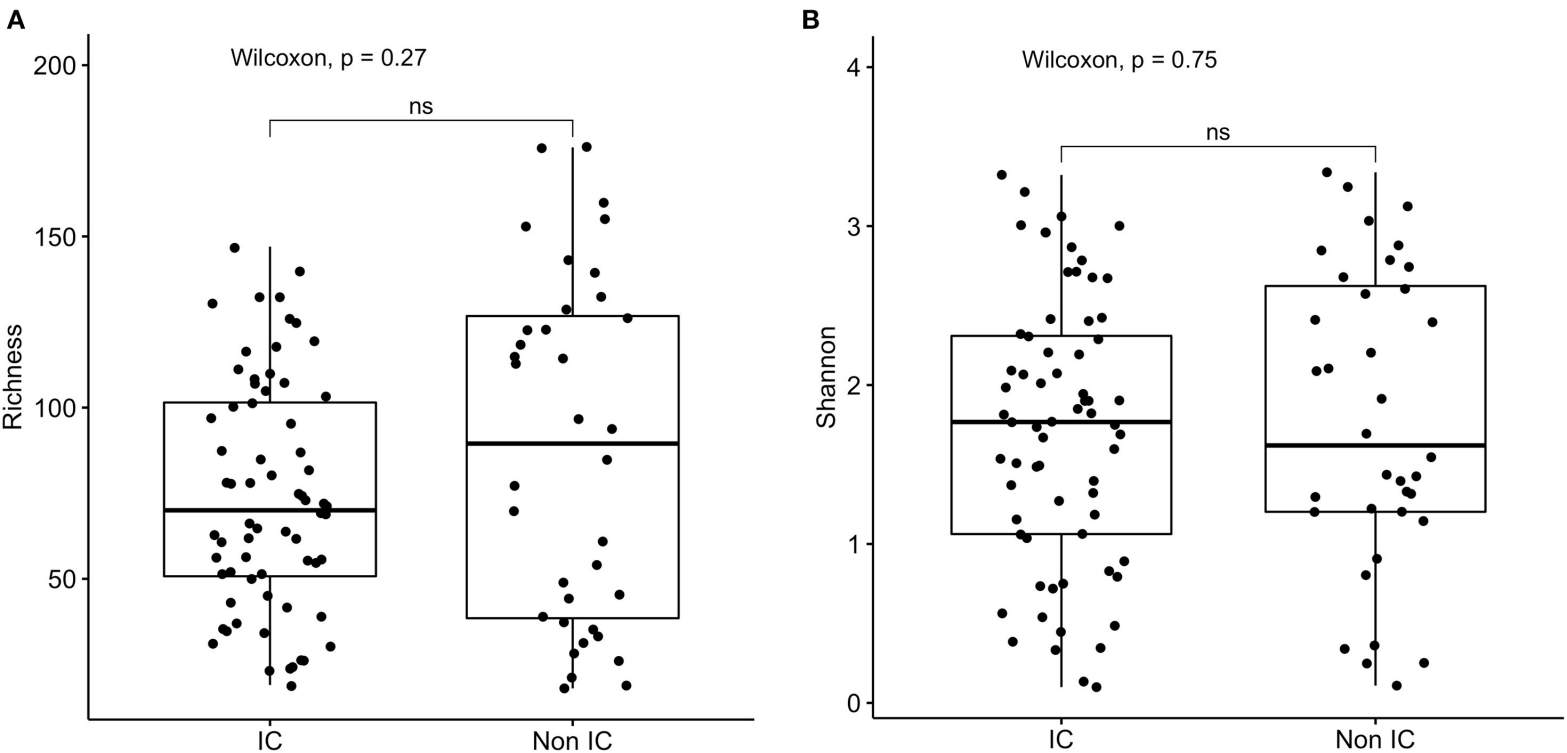
Comparison of **(A)** species richness (OTU count) and **(B)** diversity (Shannon index) comparing the IC (*n* = 69) and non-IC (*n* = 37) groups. Each point represents an individual patient and the boxplot shows the median, the first and third quartiles (bottom and top bars of the boxplot) and minimum and maximum values.

**Figure 3:**
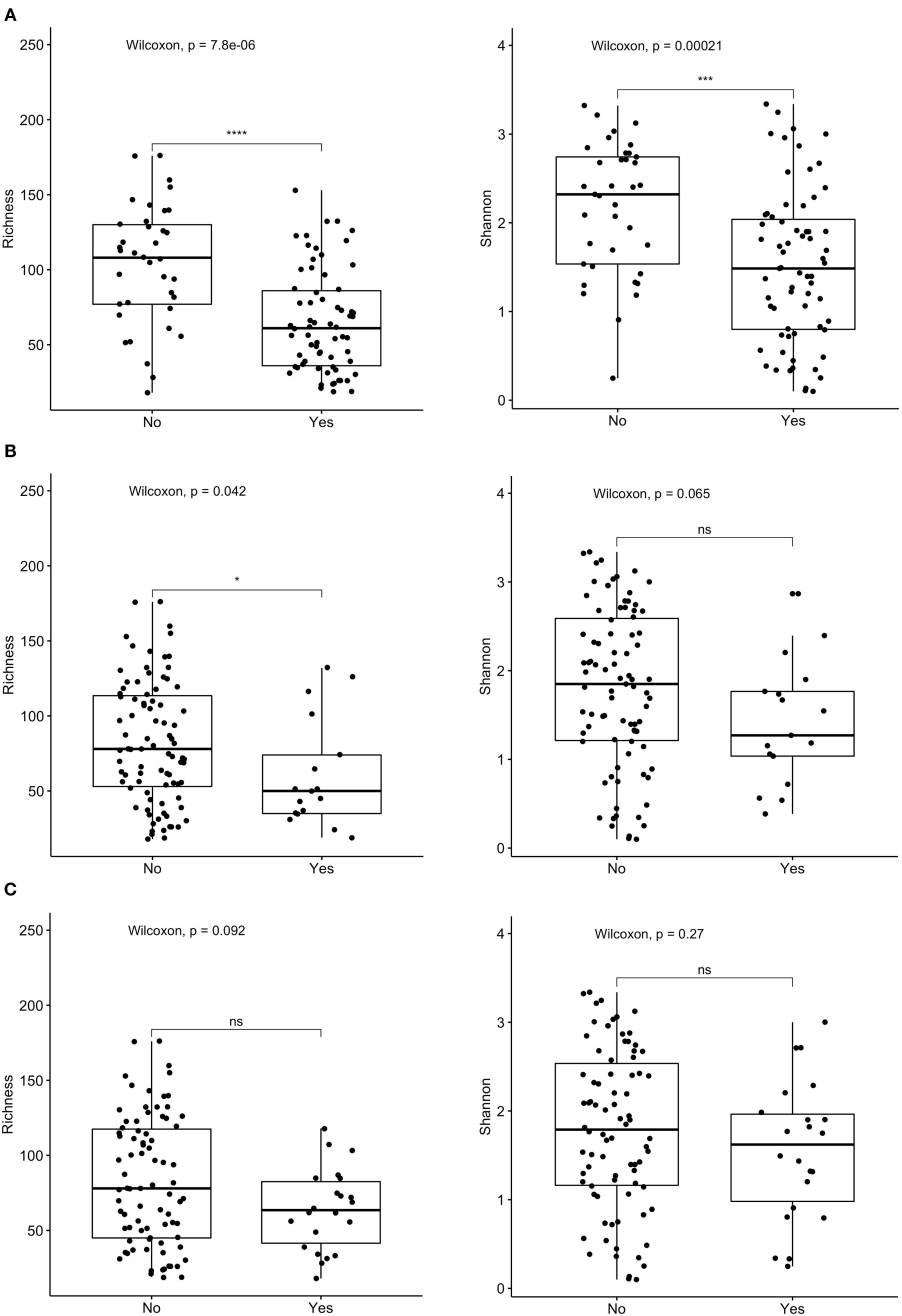
Comparison of species richness (left panel) and Shannon index (right panel) in total cohort based on receipt of **(A)** current antibiotics, **(B)** proton pump inhibitors (PPI) over the past 3 months, and **(C)** *C. difficile* colonization. ****p < 0.0001, ***p < 0.001, *p < 0.05, ns, not significant; Wilcoxon rank sum test.

16s rRNA sequencing was successfully completed in 104 participants; one non-IC participant did not have an available aliquot and one IC (SCD) sample was excluded due to poor DNA quality. Pie charts of the top 8 genera identified demonstrate that *Bacteroides* dominated overall (Figure 1). There were no significant differences in alpha diversity measured by species richness or evenness (Shannon index) comparing the IC and non-IC groups (Figures 2A,B). However, within the total population studied, current antibiotics and PPI use were associated with significantly lower species richness and evenness (Figures 3A,B). There was also a non-significant trend toward lower species richness and evenness in those who were colonized with *C. difficile* (Figure 3C).

Group dissimilarities were measured by Bray-Curtis distance considering abundance of OTUs, and by Jaccard distance assessing community membership. The overall dissimilarity did not differ between the IC and non-IC groups. However, association between variation in gut microbiota and host characteristics using these distances by Anosim (Table 2) and Adonis (Table 3), respectively, showed that gender (*p* = 0.03 and *p* = 0.039), diarrhea (*p* = 0.028 and *p* = 0.013) and PPI use (*p* = 0.003 and *p* = 0.01) were significantly associated with the gut community bacterial variation (Bray-Curtis distance). Current antibiotic use was associated with significant effects on microbial variation (*p* = 0.002, Adonis) and PPI use was associated with a significant effect on microbiome membership (Jaccard distance) in both Anosim and Adonis (*p* = 0.01 and *p* = 0.002).

**Table 2:** Analysis of similarities (ANOSIM) at the OTU level using Bray-Curtis distance and Jaccard distance to assess the similarity of bacterial communities between groups at OTU level.

	**OTUs Level (relative abundance rarefied)**			
**Variable**	**D_Bray dissimilarity**		**D_Jaccard presence/absence**	
	***R*^a^**	***p*-value**	***R*^a^**	***p*-value**
IC/Non IC	−0.035	0.84	0.0009	0.46
*C. difficile*	−0.013	0.55	0.04	0.26
Gender	0.03	0.03^*^	0.01	0.13
Abx.last 3 m	−0.007	0.75	0.009	0.15
Abx.current	−0.08	1	0.01	0.31
Diarrhea	0.18	0.028^*^	0.13	0.085
Hospitalization	0.04	0.077	0.06	0.026^*^
PPI	0.27	0.003^**^	0.19	0.01^*^

a *Correlation coefficient correlation coefficient*.

* *P ≤ 0.05*;

** *P ≤ 0.01*.

**Table 3:** Adonis analysis assessing association between variation in gut microbiota at OTU level and host characteristics using Bray-Curtis distance and Jaccard distance.

**OTUs Level (relative abundance rarefied)**						
**Variable**	**D_Bray**			**D_Jaccard**		
	**F. model^a^**	***R2*****^b^**	***p*****-value**	**F. model**	***R2***	***p*****-value**
IC/Non IC	1.03	0.01	0.41	1.18	0.011	0.16
*C. difficile*	1.28	0.013	0.082	2.34	0.022	0.001^**^
Gender	1.56	0.015	0.039^*^	1.22	0.012	0.12
Antibiotics (previous 3 mos.)	0.76	0.007	0.8	1.18	0.011	0.17
Current antibiotics	2.21	0.02	0.002^**^	3.44	0.033	0.001^**^
Diarrhea	1.89	0.018	0.013^*^	1.59	0.015	0.016^*^
Hospitalization	0.95	0.009	0.49	1.4	0.014	0.038^*^
PPI	1.95	0.019	0.01^*^	2.12	0.02	0.002^**^

a *The pseudo F ratio compares the total sum of squared dissimilarities among subjects between groups to that of subjects within group; large F-ratios indicate strong group separation*.

b *R2 value indicates the percentage of variation in the group dissimilarities explained by the variable*.

* *P ≤ 0.05*;

** *P ≤ 0.01*.

**Figure 4:**
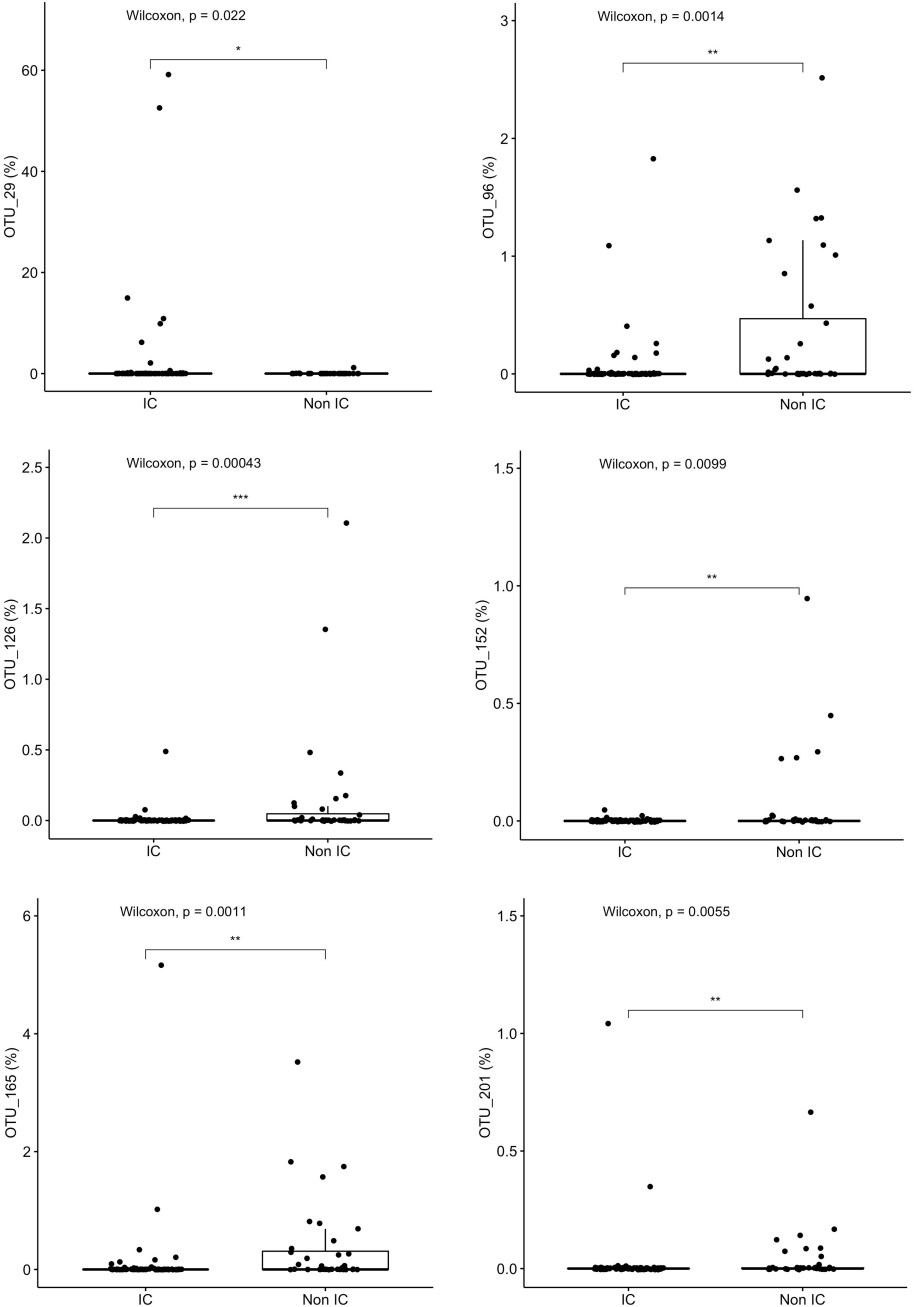
Specific species that differentiate the community composition between IC and non-IC patients identified by Random Forest plots: *Granulicatella adjacens, Roseburia inulinivorans, Roseburia intestinalis, Alistipes ihumi, Alistipes putredinis*, and *Ruminococcus albus*. ***p < 0.001, **p < 0.01, *p < 0.05, Wilcoxon rank sum test.

Variable regions one through three (V1-V3) of the 16S rRNA gene are generally sufficient to identify taxa down to the genus level and sometimes to the species level; the closest taxonomic identification based on OTU were identified and compared. *Granulicatella adjacens (OTU 29)* was more abundant in IC patients (*p* = 0.022), whereas *Roseburia inulinivorans* (OTU 165) (*p* = 0.001), *Roseburia intestinalis* (OTU 96) (*p* = 0.001), *Alistipes ihumi* (OTU 201) (*p* = 0.006), *Alistipes putredinis* (OTU 152) (*p* = 0.0099) and *Ruminococcus albus (OTU 126)* (*p* = 0.0004) were more abundant in non-IC patients (Figure 4).

Dominance of OTUs also differed when comparing participants based on PPI or antibiotic exposure. *Streptococcus parasanguinis* (OTU 55) abundance was significantly higher in those on PPI compared to those not using PPI (*p* = 0.0027). Conversely, *Eubacterium rectale (OTU 35) (p* = *0.0083), Faecalibacterium prausnitzii (OTU 38) (p* = *0.047), Anaerostipes hadrus (OTU 19) (p* = *0.029)*, and *Eggerthella lenta (OTU 131)(p* = *0.0002)* were more abundant and more prevalent in patients who were not on PPI (Supplementary Figure 1). *Bacteroides faecis* (OTU6) (*p* = 0.0076), *Paraprevotella clara* (OTU86) (*p* = 0.012), *Faecalicatena fissicatena* (OTU120) (*p* = 0.012) and *Roseburia inulinivorans* (OTU165) (*p* = 0.038) were more abundant in the patients who currently did not use antibiotics (Supplementary Figure 2). The bacteria significantly affected by antibiotics were different than those significantly affected by PPI.

### *C. difficile* Colonization

**Figure 5:**
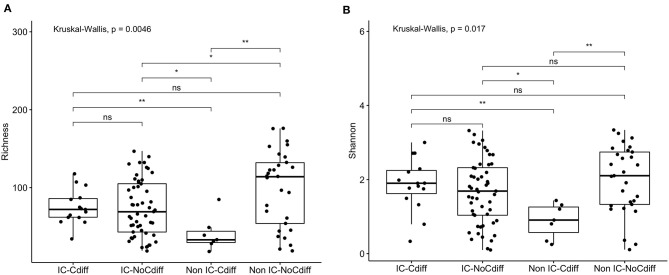
Comparison of **(A)** species richness (OTU count) and **(B)** diversity (Shannon index) comparing the IC and non-IC groups based on *C. difficile* colonization. Each point represents an individual patient and the boxplot shows the median, the first and third quartiles (bottom and top bars of the boxplot) and minimum and maximum values. **p < 0.01, *p < 0.05, ns, not significant; Wilcoxon rank sum test.

Prior studies suggest that colonization with *C. difficile*, specifically, toxigenic strains, is associated with decreased microbial diversity (17). Overall, 23/106 (22%) of the study participants were colonized with *C. difficile* (GDH assay) and the majority of these (14/23, 61%) were toxin B positive by PCR assay (Table 1). Colonization with *C. difficile* or specifically, toxin B+ *C. difficile*, did not differ comparing the IC vs. non-IC patients, or within the IC group, comparing the different subgroups. As expected, colonization was higher in children younger than 1 year of age (6/15, 40%) compared to older children (17/91, 19%) (Table 4). Non-IC, *C. difficile* colonized patients had significantly lower alpha diversity compared to non-IC, non-colonized patients (*p* < 0.01) and compared to the IC *C. difficile*-colonized group (*p* = < 0.01) (Figure 5).

**Table 4:** *C. difficile* GDH and toxin B + colonization in different subgroups of patients.

**Group**	**Any *C. difficile* [GDH, n (%)]**	**Toxin B PCR + *(% of total C. difficile)***
Age <1 year	6/15 (40)	4/6 (67)
Age > 1 year	17/91 (19)	10/17(59)
Cancer	5/19 (26)	3/5 (60%)
Post-transplantation	2/9 (22)	0
SCD	8/32 (25)	6/8 (75%)
Penicillin ppx	7/21 (33)	5/7 (71%)
No penicillin ppx	1/11 (9)	1/1 (100%)

Being colonized with *C. difficile* was associated with specific taxa, including increased proportions of *Peptoclostridium difficile* (*p* = 5e-05), *Clostridium spiroforme* (*p* = 0.064) and *Christensenella massiliensis* (*p* = 0.009) (Supplementary Figure 3). *Roseburia inulinivorans* (OTU 165) (*p* = 4.7e-05) and *Roseburia intestinalis* (OTU 96) (*p* = 0.002) were more abundant in non-IC, *C. difficile*-negative participants compared to IC or *C. difficile*-colonized patients (Supplementary Figure 4).

### Microbiome in the Subset of Children With SCD

Children with SCD, comprised the largest subgroup of the IC cohort. Comparison of this subgroup to the rest of the IC cohort and the non-IC cohort showed no significant differences in alpha diversity measured by species richness or evenness (Shannon index) (Supplementary Figure 5). The subgroup with SCD also did not differ in beta diversity (Supplementary Figure 6). However, within the SCD subset, penicillin prophylaxis was associated with a trend toward a decrease in alpha diversity (*p* = 0.11) (data not shown). Patients with SCD receiving penicillin prophylaxis had a greater abundance of *Bacteroides fragilis* (*p* = 0.035) and a lower abundance of *Bacteroides xylanisolvens* (*p* = 0.013), *C. spiroforme* (*p* = 0.0026), and *Blautia obeum* (*p* = 0.052) compared to SCD patients who were not receiving penicillin (Supplementary Figure 7). Approximately half of SCD patients were receiving hydroxyurea (16/32), which did not appear to have an impact on the bacterial composition or diversity. Notably, children with SCD receiving penicillin prophylaxis had the highest overall rate of colonization with *C. difficile* (33%) (Table 4).

## Discussion

Both measures of alpha diversity, species richness and evenness of distribution, were significantly lower in stool samples obtained from children within 72 h of hospitalization who were receiving antibiotics or PPIs. These features were found significantly more often in the population defined as IC compared to non-IC, although there were no statistically significant differences in either measure of microbial alpha diversity comparing these two groups. This may reflect the overall heterogeneity of both cohorts.

The non-IC patients, however, did have higher proportions of several bacterial species that have been associated with health including Lachnospiraceae (*Roseburia inulinivorans* and *Roseburia intestinalis*) Ruminococcaceae (*Ruminococcus albus)* and Rikenellaceae (*Alistipes ihumi* and *Alistipes putredinis*). For example, *Roseburia intestinalis* metabolize dietary fibers and produce major short-chain fatty acids, providing energy sources for enterocytes and achieving anti-inflammatory effects in the gut. The abundance of *R. inulivorans* and other butyrate producers has been shown to be decreased in patients with IBD compared to healthy controls (18). *Ruminococcus albus* is an anaerobic bacterium that produces acetate, ethanol, formate, hydrogen and carbon dioxide from carbohydrates. Hydrogen-producing *Ruminococcus albus* is more abundant in healthy individuals than in patients with Crohn's disease (19) and has probiotic effects (20). Similarly, *Alistipes* species characterize a healthy gut microbiome and a recent study of newly diagnosed pediatric patients with IBD or Crohn's disease found that IBD was characterized by decreased abundance of *Alistipes finegoldii* and A*listipes putredinis* (21).

The observation that current antibiotic use is associated with decreased diversity is consistent with numerous studies in the literature (22–25). We also observed that within the subgroup of IC patients with SCD, those on penicillin prophylaxis trended toward having lower alpha diversity, higher rates of asymptomatic *C. difficile* colonization, and differences in proportions of select bacteria. Specifically, SCD patients receiving penicillin had a lower proportion of Lachnospiraceae, a family of probiotic, anaerobic bacteria, including *Blautia obeum* and *Bacteroides xylanisolvens*. These bacteria play a major role in the fermentation and degradation of plant fibers, producing short-chain fatty acids with potential health-beneficial effects and may also possess immunomodulatory properties (26). These differences in microbiome among children with SCD receiving penicillin prophylaxis may also be impacted by age since prophylaxis is typically prescribed only in children younger than 5 years. SCD patients on penicillin were significantly younger (mean ± SEM 3.9 ± 0.93 years) than those not on penicillin (mean ± SEM 9.69 ± 0.89 years) (*p* < 0.007), but age-adjusted analysis could not be performed in the current study due to the small sample size.

Few studies have described the microbiome in patients with SCD. Microbial dysbiosis has been described in two small studies of adults with SCD (27, 28) and in one was associated with higher lactate dehydrogenase levels, which may be a biomarker of ongoing hemolysis (28). However, the link between the gut microbiome and SCD is complex. For example, depletion of microbiota with broad-spectrum antibiotics reduced the number of aged neutrophils and protected against organ damage in a murine model of SCD (29). Thus, further studies are needed to determine the clinical implications of changes in gut microbiome associated with SCD and with penicillin prophylaxis in this population.

PPIs were also associated with significant decreases in alpha diversity and microbial composition (beta diversity). Concerns for PPI use and its effect on the gut microbiome and risk for *C. difficile* colitis have been raised in recent years (30–33) but the effects on microbial diversity and, specifically, in pediatric patients have not been well-studies. A recent Japanese study comparing 36 adults taking PPI vs. 36 healthy controls found no differences in alpha diversity but did find differences in microbial composition; *Streptococcus* was significantly more abundant and *Faecalibacterium* significantly decreased in PPI users (34). In a larger adult twin study, PPI use was associated with decreased alpha diversity and increases in Streptococcaceae and other oral or upper GI tract flora, suggesting that removal of the low pH barrier between the upper and lower tract contributes to the observed differences (31). We also observed significant increases in *Streptococcus parasanguinis* and decreases not only in *Faecalibacterium prausnitzi*, but also in *Eubacterium rectale, Anaerostipes hadrus* and *Eggerthella lenta* in our cohort of PPI users. However, the clinical consequences of these microbial composition differences require further study.

Nearly one-quarter of hospitalized pediatric patients were found to have asymptomatic *C. difficile* colonization and the majority of these were toxin B+, only by PCR; none were EIA positive. This phenotype (GDH-positive, EIA toxin-negative, PCR+) is well-described in studies of colitis, but not colonization, and is associated with less severe disease and fewer recurrences compared to patients who have colitis associated with EIA toxin + isolates (35). Antibiotic and PPI use have been shown to be risk factors for *C. difficile* infection in children (36). However, there are relatively fewer studies on colonization. Consistent with prior studies, *C. difficile* colonization was positively associated with younger age and with recent antibiotic exposure (37, 38). Colonization was also associated with a decrease in microbial evenness (Shannon index) and, within the non-IC cohort, decreased richness, consistent with findings of decreased gut microbial diversity in patients with *C. difficile* colitis (39, 40).

The non-IC cohort in general and, specifically those who were not colonized with *C. difficile*, had higher proportions of Lachnospiraceae (*Roseburia*), which are involved in the production of butyrate. Butyrate is the preferred energy source of colonic endothelial cells (colonocytes) and plays a major role in the regulation of colonocyte proliferation and the differentiation and maintenance of intestinal epithelial integrity (41). Lachnospiraceae were shown to be partially protective against *C. difficile* in murine and human studies (42–44). For example, in adult allogeneic-HSCT patients, Lachnospiraceae were associated with a 60% lower risk of *C. difficile* colitis independent of other risk factors (44).

There are several limitations to this cross-sectional, single-center study including the diverse nature of the IC cohort, lack of longitudinal samples and age differences in the study populations, particularly comparing children with SCD who were or were not receiving penicillin prophylaxis. It is also known that genetic factors including HLA type can influence the microbial composition, which could account for some of the differences in the groups (45).Despite these limitations, the results have generated several hypotheses that should be addressed in future larger, longitudinal studies. In addition to the findings that antibiotics and PPIs are associated with decreased microbial diversity and changes in species composition, the study uncovered a potentially previously unrecognized patient population at risk for an altered gut microbiome: children with SCD, particularly, those receiving penicillin prophylaxis. Understanding the implications of the observed differences in the microbiome in this population may have implications for the future management of patients with SCD.

## Data Availability Statement

The original contributions presented in the study are publicly available. This data can be found at: https://www.ebi.ac.uk/ena/browser/view/PRJEB40113.

## Ethics Statement

The Albert Einstein College of Medicine-Montefiore Institutional Review Board approved the study. Written informed consent to participate in this study was provided by the participants' legal guardian/next of kin.

## Author Contributions

SM contributed to conception and design, data collection and interpretation, sample collection and testing, drafted the initial manuscript, and conducted the literature review. TA participated in patient recruitment, data collection, sample collection, and testing and edited the document. TDBT, ES, and GW contributed to the microbiome sequencing, analysis, interpretation, discussion, and edited the document. VS and DG contributed in drafting the manuscript and editing the document. ES, GW, and BH critically reviewed the manuscript for important intellectual content and provided supervision. All authors provided critical feedback, helped shape the research, analysis, manuscript, and approved the final manuscript as submitted and agree to be accountable for all aspects of the work.

## Conflict of Interest

The authors declare that the research was conducted in the absence of any commercial or financial relationships that could be construed as a potential conflict of interest.

## Supplementary Material

Supplementary Figure 1 Specific species that differentiate the community composition between patients who were on PPI (Yes) compared to those who were not (No), identified by Random Forest plots: *Streptococcus parasanguinis, Eubacterium rectale, Faecalibacterium prausnitzii, Anaerostipes hadrus and Eggerthella lenta*. ^***^*p* < 0.001, ^**^*p* < 0.01, ^*^*p* < 0.05, Wilcoxon rank sum test.

Supplementary Figure 2 Specific species that differentiate the community composition between patients based on if they were currently on antibiotics (Yes) or not (No), identified by Random Forest plots: *Bacteroides faecis, Paraprevotella clara, Faecalicatena fissicatena, Roseburia inulinivorans*. ^**^*p* < 0.01, ^*^*p* < 0.05, Wilcoxon rank sum test.

Supplementary Figure 3 Random Forests used to identify specific species *Peptoclostridium difficile, Clostridium spiroforme* and *Christensenella massiliensis* that differentiate the community composition based on *C. difficile* colonization status (Yes) or (No). ^****^*p* < 0.0001, ^***^*p* < 0.001, ^*^*p* < 0.05, ns, not significant; Wilcoxon rank sum test.

Supplementary Figure 4 Random Forests used to identify specific species *Roseburia inulinivorans* and *Roseburia intestinalis* that differentiate the community composition between IC and non-IC patients based on their *C. difficile* colonization status (“C diff” vs. “No C diff”). ^***^*p* < 0.001, ^**^*p* < 0.01, ^*^*p* < 0.05, ns, not significant; Kruskal- Wallis test.

Supplementary Figure 5 Comparison of **(A)** species richness (OTU count) and **(B)** diversity (Shannon index) comparing the SCD (*n* = 32), IC (without SCD) (*n* = 37) and non-IC (*n* = 37) groups. Each point represents an individual patient and the boxplot shows the median, the first and third quartiles (bottom and top bars of the boxplot) and minimum and maximum values.

Supplementary Figure 6 PCoA plot: SCD samples do not cluster differently from samples in other cohorts.

Supplementary Figure 7 Random Forests used to identify specific species (*Bacteroides xylanisolvens, Bacteroides fragilis, Clostridium spiroforme* and *Blautia obeum*) that differentiate the community composition between SCD patients, based on if they were receiving penicillin prophylaxis (Yes PCN) or not (No PCN). ^**^*p* < 0.01,^*^*p* < 0.05, ns, not significant; Wilcoxon rank sum test.
