# Supplementary material for: Differences in Gut Microbiome in Hospitalized Immunocompetent vs. Immunocompromised Children, Including Those With Sickle Cell Disease
Source: Front Pediatr. 2020 Nov 12;8:583446. doi: 10.3389/fped.2020.583446 (PMC7690629; doi:10.3389/fped.2020.583446)
Supplement: Supplementary file 1 [file Table_1.docx]

Supplemental Table1. Diagnoses in Immunocompromised and Non-immunocompromised groups

| 1. **Immunocompromised group** | N |
| --- | --- |
| Sickle cell disease | 32 |
| Malignancy | 19 |
| Post liver or renal transplant | 6 |
| Post human stem cell transplant | 3 |
| Nephrotic syndrome and high dose steroids | 3 |
| Chronic granulomatous disease | 1 |
| Shwachman-Diamond syndrome | 1 |
| Pancytopenia | 1 |
| Neutropenia | 1 |
| Autoimmune thrombocytopenia and high dose steroids | 1 |
| Liver failure, vasoocculsive disease, high dose steroids | 1 |
| 1. **Non Immunocompromised group** |  |
| Post-surgical | 14 |
| Pyelonephritis | 2 |
| Abdominal mass | 2 |
| Hemophilia or Von Willebrand Disease | 4 |
| Acute autoimmune thrombocytopenia (no steroids) | 2 |
| Concussion | 1 |
| Left humeral mass | 1 |
| Abdominal pain and vomiting | 1 |
| Gastritis | 1 |
| Viral syndrome | 1 |
| Biliary atresia | 1 |
| Thrombocytopenia evaluation | 1 |
| Intracranial hemorrhage | 1 |
| Asthma | 1 |
| Ventriculoperitoneal shunt infection/obstruction | 2 |
| Common biliary duct stricture | 1 |
| Facial nerve palsy | 1 |
