# Supplementary figures and images for: Differences in Gut Microbiome in Hospitalized Immunocompetent vs. Immunocompromised Children, Including Those With Sickle Cell Disease
Source: Front Pediatr. 2020 Nov 12;8:583446. doi: 10.3389/fped.2020.583446 (PMC7690629; doi:10.3389/fped.2020.583446)

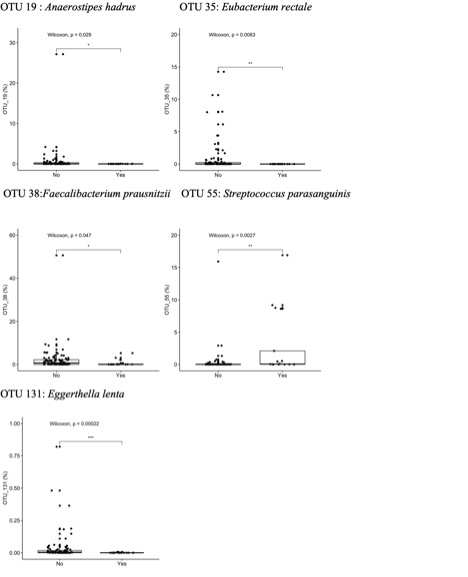

Supplement: Supplementary Figure 1 — Specific species that differentiate the community composition between patients who were on PPI (Yes) compared to those who were not (No), identified by Random Forest plots: Streptococcus parasanguinis, Eubacterium rectale, Faecalibacterium prausnitzii, Anaerostipes hadrus and Eggerthella lenta. ***p < 0.001, **p < 0.01, *p < 0.05, Wilcoxon rank sum test. [file Image_1.JPEG]

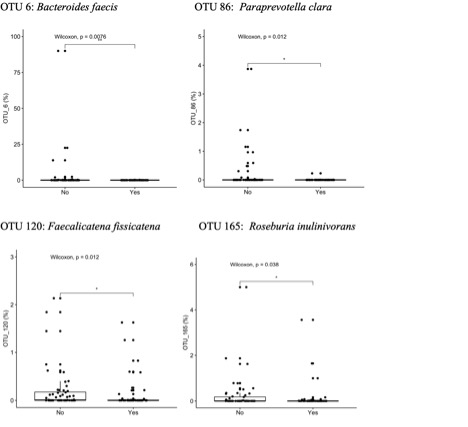

Supplement: Supplementary Figure 2 — Specific species that differentiate the community composition between patients based on if they were currently on antibiotics (Yes) or not (No), identified by Random Forest plots: Bacteroides faecis, Paraprevotella clara, Faecalicatena fissicatena, Roseburia inulinivorans. **p < 0.01, *p < 0.05, Wilcoxon rank sum test. [file Image_2.JPEG]

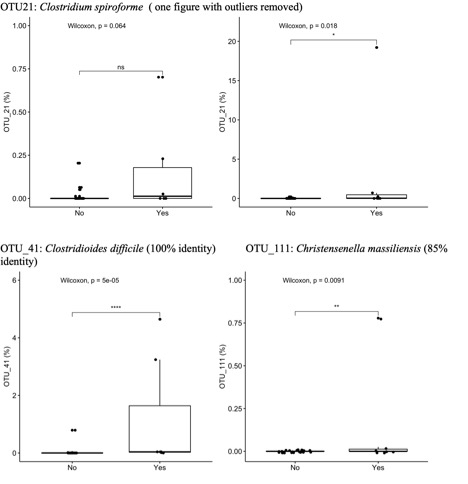

Supplement: Supplementary Figure 3 — Random Forests used to identify specific species Peptoclostridium difficile, Clostridium spiroforme and Christensenella massiliensis that differentiate the community composition based on C. difficile colonization status (Yes) or (No). ****p < 0.0001, ***p < 0.001, *p < 0.05, ns, not significant; Wilcoxon rank sum test. [file Image_3.JPEG]

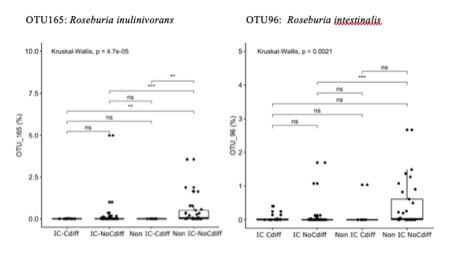

Supplement: Supplementary Figure 4 — Random Forests used to identify specific species Roseburia inulinivorans and Roseburia intestinalis that differentiate the community composition between IC and non-IC patients based on their C. difficile colonization status (“C diff” vs. “No C diff”). ***p < 0.001, **p < 0.01, *p < 0.05, ns, not significant; Kruskal- Wallis test. [file Image_4.JPEG]

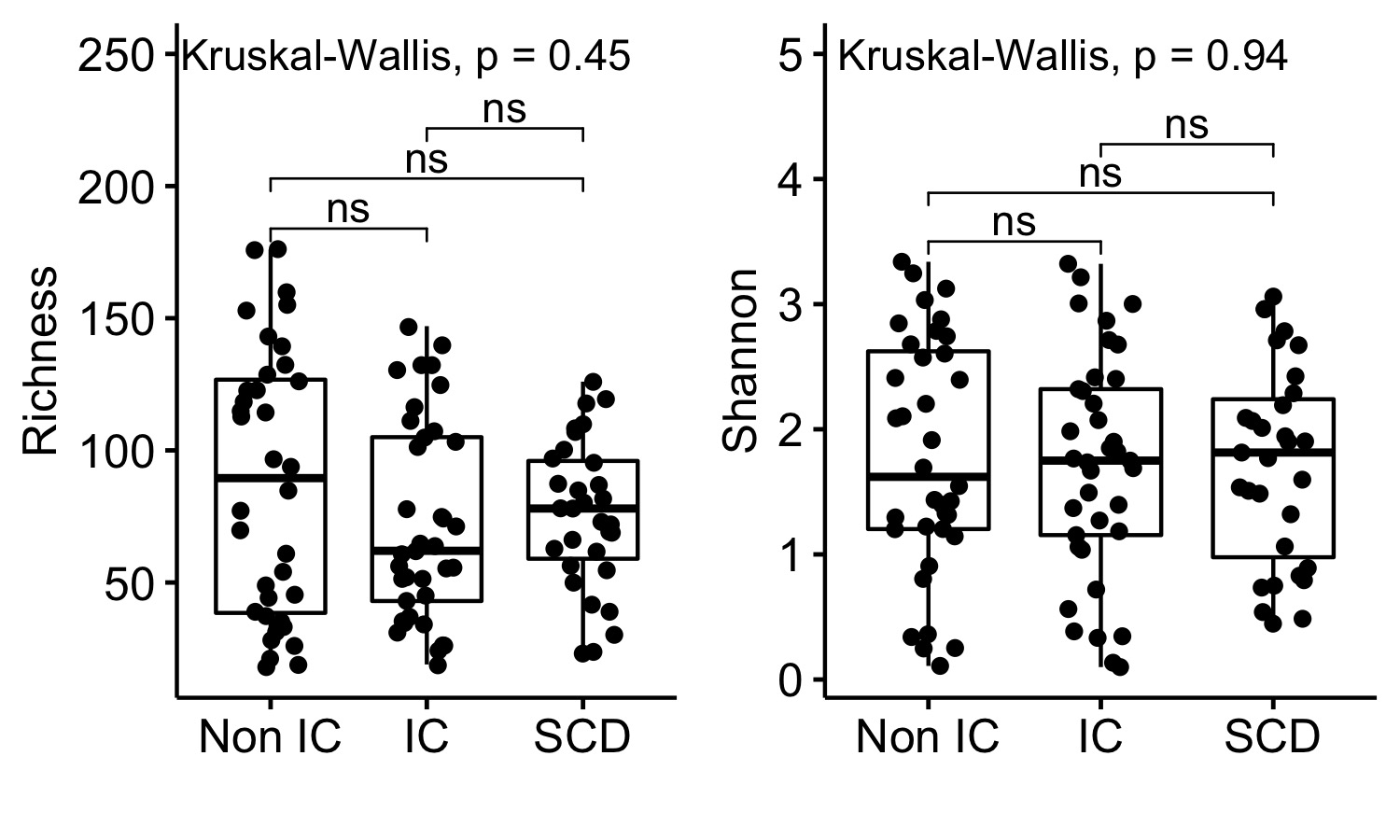

Supplement: Supplementary Figure 5 — Comparison of (A) species richness (OTU count) and (B) diversity (Shannon index) comparing the SCD (n = 32), IC (without SCD) (n = 37) and non-IC (n = 37) groups. Each point represents an individual patient and the boxplot shows the median, the first and third quartiles (bottom and top bars of the boxplot) and minimum and maximum values. [file Image_5.JPEG]

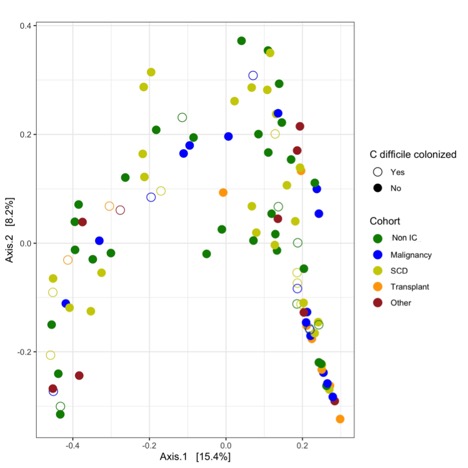

Supplement: Supplementary Figure 6 — PCoA plot: SCD samples do not cluster differently from samples in other cohorts. [file Image_6.JPEG]

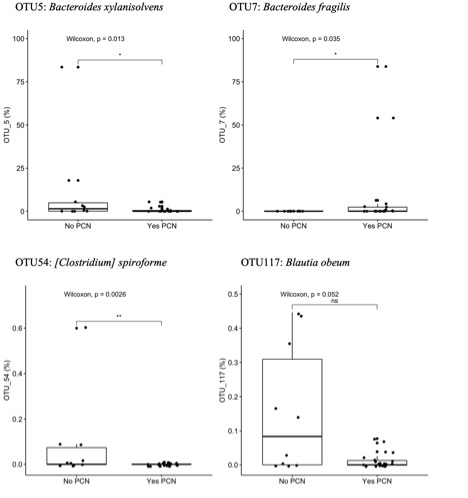

Supplement: Supplementary Figure 7 — Random Forests used to identify specific species (Bacteroides xylanisolvens, Bacteroides fragilis, Clostridium spiroforme and Blautia obeum) that differentiate the community composition between SCD patients, based on if they were receiving penicillin prophylaxis (Yes PCN) or not (No PCN). **p < 0.01,*p < 0.05, ns, not significant; Wilcoxon rank sum test. [file Image_7.JPEG]
